# A common self-advantage across the implicit and explicit levels for self-body recognition

**DOI:** 10.3389/fpsyg.2023.1099151

**Published:** 2023-08-11

**Authors:** Sijia Xiang, Minghui Zhao, Lunhao Yu, Ning Liu

**Affiliations:** ^1^State Key Laboratory of Brain and Cognitive Science, Institute of Biophysics, Chinese Academy of Sciences, Beijing, China; ^2^Sino-Danish College, University of Chinese Academy of Sciences, Beijing, China; ^3^College of Life Sciences, University of Chinese Academy of Sciences, Beijing, China; ^4^Institute of Artificial Intelligence, Hefei Comprehensive National Science Center, Hefei, China

**Keywords:** self-body, hand, explicit, implicit, self-bias

## Abstract

**Introduction:**

Although self-bias has been extensively studied and confirmed in various self-related stimuli, it remains controversial whether self-body can induce recognition advantage at the explicit level. After careful examination of previous experiments related to self-body processing, we proposed that participant strategies may influence explicit task outcomes.

**Methods:**

To test our hypothesis, we designed a novel explicit task. For comparison, we also conducted classic explicit and implicit tasks.

**Results:**

With the newly designed explicit task, we found clear and robust evidence of self-hand recognition advantage at the explicit level. Moreover, we found that there was a strong link between self-advantage found in the classic implicit task and the newly designed explicit task, indicating that the self-advantage processing by these two pathways may be linked.

**Discussion:**

These findings provide new insights into the long-standing inconsistencies in previous studies and open a new avenue for studying self-bias using self-body stimuli.

## 1. Introduction

Self-consciousness is the basis of higher cognitive functions in humans. It is an essential concept in philosophy, psychology, and cognitive neuroscience. Self-consciousness serves as a framework to understand how individuals interact with the world and contemplate both their own behavior and the behavior of others. Within this framework, the self-system processes self-relevant information and adjusts the impact of self-consciousness on cognitive processes, considering factors such as goal sensitivity (Cunningham and Turk, 2017). Previous studies have suggested that the perpetual goal of the self-system is to ensure that self-relevant information can be preferentially processed (Cunningham and Turk, 2017). This preference for self-relevant information manifests as systematic biases in perception, memory, and attention, where individuals tend to prioritize information that pertains to the self over information related to others. This phenomenon is called self-bias (Sui and Humphreys, 2015, 2017; Cunningham and Turk, 2017). Therefore, self-bias serves as a manifestation of self-consciousness (Sui and Humphreys, 2017). Self-bias can also lead to more accurate processing of familiar people, enabling better inference of their emotions and thoughts in a given situation (Nijhof et al., 2020). To date, evidence of self-bias has been provided in a variety of areas, including perception (Sui et al., 2012; Sui and Humphreys, 2015, 2017), memory (Leshikar et al., 2015; Andrews et al., 2020), and attention (Yang et al., 2013; Humphreys and Sui, 2016).

Although self-bias has been extensively studied and confirmed in self-related stimuli, such as self-face (Sugiura et al., 2008; Malaspina et al., 2018, 2019), self-name (Sugiura et al., 2008; Yang et al., 2013), self-associated geometric shapes (Sui et al., 2012), and self-appraisal (Yue et al., 2021), whether the self-body, a fundamental part of self-consciousness, can induce recognition advantage remains controversial. Several studies have reported that self-hand stimuli are processed faster and more accurately than the hand images of other individuals in implicit tasks (Frassinetti et al., 2011; Richetin et al., 2012; De Bellis et al., 2017; Malaspina et al., 2019). However, this kind of self-advantage has not been found (Frassinetti et al., 2011; Richetin et al., 2012) and even reversed to a self-disadvantage (Ferri et al., 2011; Conson et al., 2015; Candini et al., 2016; Fukui et al., 2020) when participants are required to explicitly make “own hand” or “another person’s hand” judgments. Thus, it has been proposed that there is a possible dissociation between implicit and explicit bodily self-processing. To date, few studies have directly investigated such a possible dissociation, and the findings remain inconclusive. Experiments in patients with brain injury seem to provide anatomical evidence supporting such dissociation, with different areas of the right hemisphere underpinning implicit and explicit self-body knowledge (Candini et al., 2016). However, one neuroimaging study found that explicit and implicit self-processing recruit similar neural networks (Rameson et al., 2010). Recently, Conson et al. (2017) observed self-advantage in both implicit and explicit tasks by adding hand stimuli in palm view. They proposed that palm views, which have a high sensorimotor load, may enhance motor imagery engagement and sensorimotor processing to generate self-advantage. However, it should be noted that they also found self-advantage in back views as well as palm views of the hands. Thus, these findings cannot fully explain the controversial results in implicit and explicit self-body recognition. More recently, based on meta-analysis and experimental evidence, Holmes and colleagues even suggested that hand recognition may have no self-advantage (Holmes et al., 2022). Thus, it remains unclear whether and how self-advantage exists in body recognition.

Notably, in contrast to the observed inconsistency in self-body bias, self-advantage has been found in both implicit and explicit self-face recognition in healthy participants (Devue et al., 2007; Keyes and Brady, 2010; Ma and Han, 2010) and in patients with congenital prosopagnosia (Malaspina et al., 2018, 2019), even in the same explicit self-discrimination task (e.g., Malaspina et al., 2018) as for self-body recognition (e.g., Ferri et al., 2011). These findings indicate that the inconsistency between implicit and explicit tasks does not persist uniformly across different self-related domains. Since self-face and self-body are both fundamental components of self-consciousness, it is crucial to thoroughly examine the inconsistencies in self-body bias to enhance our understanding of the underlying mechanisms of self-bias.

It has been demonstrated that body recognition is a critical self-recognition element (Gillihan and Farah, 2005). Self-body recognition may prove particularly beneficial when primary sources of one’s identity (e.g., the face) are inaccessible (Frassinetti et al., 2010). Especially, the self-body recognition paradigm can be applied to animals, which may have never seen their own faces but possess a relatively detailed visual representation of their anterior extremities (Heschl and Burkart, 2006). Therefore, self-body recognition allows for the exploration of the evolutionary origin of self-awareness. Additionally, in comparison to faces, bodies exhibit less variability; as such, self-body recognition could offer a more generalized method for assessing self-bias and may even help quantify the ability to distinguish oneself from others (Jenkins et al., 2011). Therefore, it is important to resolve the debate associated with the self-body to elucidate the extent to which self-bias is conserved across different self-related information and across implicit and explicit measures.

After carefully examining previous explicit and implicit experiments related to self-body processing, we proposed that participants’ strategies in explicit tasks may contribute to the previously confused findings at the explicit level. In classic implicit tasks, participants are usually required to choose the stimuli that match the target. That is, participants need to observe the target first, compare the choices to the target, and then make a decision. If the target is the self-hand, it may facilitate observation and comparison, and thereby impact subsequent decisions. Therefore, it is not surprising that self-advantage is reported in such implicit tasks (Frassinetti et al., 2011; Richetin et al., 2012; Malaspina et al., 2019). On the other hand, in classic explicit tasks (i.e., self-other discrimination task), participants are required to judge whether the hand image displayed on the screen is their own or another’s. If participants perform the task by determining whether the hand on screen belongs to them (referred to as “Self” judgment) or someone else (referred to as “Other” judgment), self-advantage, if it exists, would facilitate faster identification of their own hands compared to identifying others’, vice versa. However, it should be noted that participants are unlikely to make an “Other” judgment based on recognizing an unfamiliar hand. In other words, when participants make an “Other” judgment, they may also rely on whether they can identify the hand on the screen as their own. That is, both positive (resulting in a “Self” judgment) and negative responses (resulting in an “Other” judgment) may be made based on identifying the “Self.” As such, the designed investigation in classic explicit tasks, whether there is a difference between identifying the own and other’s hand at the explicit level, actually becomes whether there is a difference between positive and negative judgments of identifying the “Self.” Generally, it takes longer to make a “yes” decision than a “no” decision (Mynatt et al., 1977). Additionally, in hand recognition tasks, previous studies have suggested that participants may pay more attention to local details compared to processing faces holistically (Conson et al., 2017; Kuroki and Fukui, 2020). Consequently, once participants identify a local feature that does not belong to themselves, they can quickly choose the negative (“Other”) response. However, they may need multiple clues (features) to confirm that the images represent their own hands and then make the positive (“Self”) decision since others’ hands may also display one or more similarities to their own hands. Indeed, research has shown that participants are more likely to make “not their own” judgments, especially when the task is difficult, e.g., with shorter presentation times (Holmes et al., 2022). In this context, the advantage of making a “no” decision (recognizing the hand on the screen as not one’s own) leads to the advantage for “other” judgments, which may be interpreted as self-disadvantage in the explicit task.

In the present study, we hypothesized that the inconsistency between self-body bias in explicit and implicit tasks may be related to behavioral strategies in explicit tasks. To overcome this strategy issue, we aimed to design a novel explicit task in which participants are required to judge the identity of both “Self” and “Other” rather than only determining whether the hand image on the screen is their own or not. For comparison, we also conducted classic explicit and implicit tasks. We further performed two additional modified explicit tasks based on classic tasks to eliminate the potential influence of stimuli richness. Based on our novel explicit task, we expected that self-hand recognition advantage existed in the explicit task as well as in the implicit task.

## 2. Methods

### 2.1. Participants

Given the within-subjects design conducted in the present study, the sample size was determined based on the desired power (0.80), alpha level (0.05), effect size (0.40), and the number of measurements (four in Experiments 1 and 3, two in Experiments 2 series) before the study. Using G*Power 3.1 (Faul et al., 2007), the minimum required sample sizes were calculated as 10 for Experiments 1&3 and 15 for Experiments 2 series.

The present study consisted of 32 adult participants, including 16 males (18–25 years, mean (M) ± standard deviation (SD) = 22.88 ± 2.31 years) and 16 females (23–26 years, M = 23.88 ± 1.15 years). Two participants were excluded because they failed the visual familiarity test, and one participant was excluded due to excessive hand focus from long-term piano practice. Participants S1-S13 only performed Experiments 1, 2a, and 3. Participants S14-S29 completed all five experiments, with four of them did not perform Experiment 2a due to technical issues and one participant identified as an outlier in Experiment 2a due to her performances exceeding 2.5 SD from the mean. Since we did not try to compare the degree of self-bias across experiments, we used data from all the participants, including those who did not complete all experiments, to thoroughly verify our hypothesis and ensure the robustness of our findings. Note that the data from the same participants were utilized for the correlation analyzes conducted across experiments. Details of participants for each experiment are given in Table 1. Participants reported no abnormal neurological history, had the normal or corrected-to-normal vision, and were right-handed. All participants provided written informed consent prior to the experiment and were compensated for their participation. All procedures were approved by the Institutional Review Board (2018-IRBH-001).

**Table 1:** Demographic details of participants in five experiments.

Exp	*N*	F	Age	Private SCS	Public SCS	SAS	SES
1&3	29 (S01–S29)	15	23.38 (1.92)	25.69 (3.22)	19.93 (4.43)	12.66 (4.91)	33.31 (4.71)
2a	24 (S01–S24)	12	23.25 (2.03)	25.50 (3.39)	19.96 (4.22)	11.83 (4.90)	34.38 (3.55)
2b&2c	16 (S14–S29)	10	23.31 (1.14)	24.94 (3.07)	19.38 (3.96)	14.75 (3.73)	31.31 (4.6)

Data of age and scores in questionnaires are expressed as mean (SD).

Exp, experiment; N, number of participants; F, number of female participants; SCS, self-consciousness score; SAS, social anxiety score; SES, self-esteem score; SD, standard deviation.

### 2.2. Stimuli

**Figure 1:**
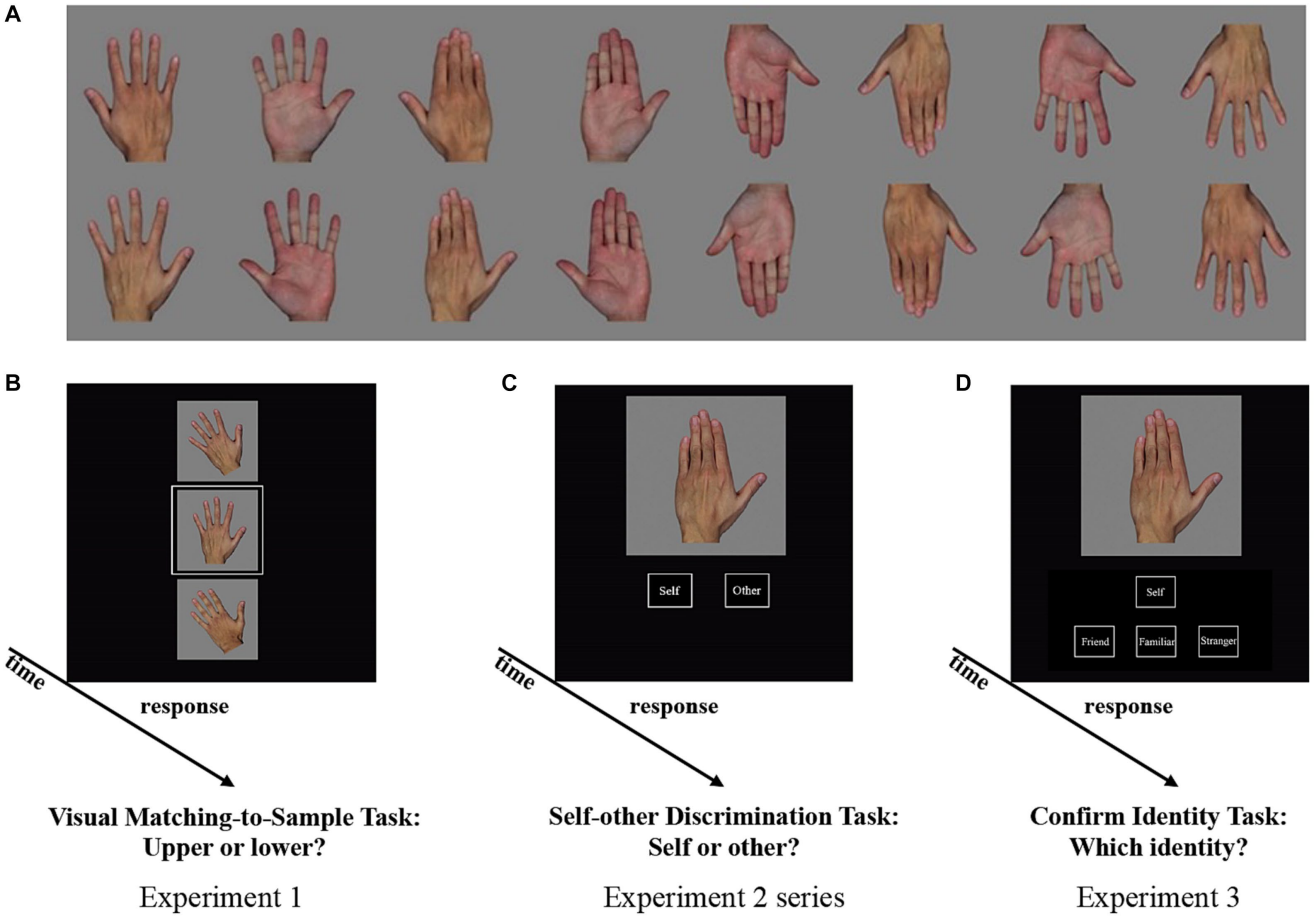
Experimental design. **(A)** Examples of stimuli used in the study. In total, 16 hand images (two hands [left or right] × two views [back or palm] × two gestures [closed or open] = eight upright images and 180° rotated versions) from each participant were used in the experiments. **(B)** Procedures of Experiment 1. Three stimuli depicting the same hand gesture were simultaneously presented vertically in the middle of the screen in each trial. The central stimulus (target) was surrounded by a white frame. Participants were required to judge which of the two images (upper or lower) matched the central target. **(C)** Procedures of Experiment 2 series (2a, 2b, and 2c). One hand image was presented on the screen. Participants were required to judge whether the displayed hand was their own or others’. **(D)** Procedures of Experiment 3. One hand image was presented on the screen. Participants were required to judge whether the displayed hand corresponded to their own, their friend, visually familiar individual, or stranger using four arrow keys.

Three days before the experiments, hand images of each participant and their same-sex friends were taken in a controlled environment with constant artificial light and a fixed distance (80 cm) between the hand and camera. Color photographs of the back and palm views of the left and right hands with closed and open gestures were taken for each participant, their friend, one visually familiar individual, and one or two strangers used as the main experimental stimuli, with an additional stranger set as the distractor in the visual familiarity test. All non-self images of hand were from individuals of the same sex as the participant. Hand images of strangers were selected from other participants unknown to the participant. To elucidate the role of visual familiarity in the observed self-bias effect, we chose one of the strangers as the “visually familiar individual,” which referred to individuals with whom participants only became visually familiar through the hand images presented on the screen without physical contact (Bola et al., 2021). Hand images were processed using Adobe Photoshop (Adobe Systems, San Jose, CA, United States) to remove the background. Low-level image properties (mean luminance and contrast) were then equated across stimulus categories (i.e., self, friend, visually familiar individual, and strangers) for each participant using the SHINE toolbox (Willenbockel et al., 2010). Finally, for each participant, 16 images (two hands [left or right] × two views [back or palm] × two gestures [closed or open] = eight upright and 180° rotated versions) were created for each of the four categories, as shown in Figure 1A.

In Experiments 1 (Visual Matching-to-Sample Task, the classic implicit task), 2c (Self-Other Discrimination Task, the classic explicit task), and 3 (Identity Discrimination Task, the newly designed explicit task), 16 hand images from each of the four categories (self, friend, visually familiar individual, and stranger) were presented (n = 64). Only one stranger was used in these three experiments.

In Experiments 2a and 2b (Self-Other Discrimination Task, the classic explicit task), only stimuli from self and strangers were presented. To avoid ceiling effects, eight and 16 hand images from each of the two strangers were used in Experiments 2a and 2b, respectively. Sixteen self-hand images were used in Experiments 2a and 2b.

### 2.3. Procedure

Experiments were conducted using Matlab 2016a (MathWorks, Natick, MA, United States) and PsychToolbox (Brainard, 1997).^1^ Participants were seated in a comfortable chair in a sound-attenuated room. All stimuli were presented against a uniform black background on a 22-inch monitor (Dell P2217H, 1920 × 1,080 pixels). When viewed from 68 cm, the hand images subtended a visual angle of 6.41° (H) × 6.41° (W) in the implicit experiment and 7.31° (H) × 7.31° (W) in the explicit experiments.

The experiments started with a brief preview phase. To ensure that the image processing procedures, specifically the cropping and matched brightness of the images, did not result in participants being unable to recognize their own and their friend’s hands, we implemented a brief preview phase as a precautionary measure before the experiments. During this phase, participants were presented with processed images of their own and their friends’ hands (16 images from each identity) in a random order, utilizing a rapid visual presentation format with a speed of 800 ms per image. Subsequently, participants were asked to tell the experimenters whose hands they had seen. All participants reported seeing images of their own and their friend’s hands, indicating that processed images could be recognized. As such, no participants were excluded due to failure to pass the brief preview phase. It is essential to emphasize that during this phase, participants were not required to identify the specific identity associated with each stimulus. Next, participants were given as much time as needed to become familiar with eight upright hand images of the visually familiar individual. A visual familiarity test was performed using 32 hand images from the visually familiar individual and a distractor (stranger) to ensure that participants were able to recognize hand images from the visually familiar individual. Each hand image was presented for 1 s, and participants were required to explicitly judge whether the displayed hand corresponded to the visually familiar individual or not. If the judgment was correct, the next image was presented. If not, the hand image with the same gesture from the visually familiar individual was presented again for re-familiarization. When participants reached the criterion of an 80% correct rate, they moved to the test phase.

In Experiment 1, three stimuli depicting the same hand gesture were simultaneously presented vertically in the middle of the screen in each trial (shown in Figure 1B). The central stimulus (target) was surrounded by a white frame. Participants were required to judge which of the two images (upper or lower) matched the central target. To minimize automatic matching between stimuli, the upper and lower stimuli were tilted 15° to the left or right relative to the central target. There were four conditions in Experiment 1: *Self*, *Friend*, *Visually Familiar Individual*, and *Stranger*. Experiment 1 consisted of 384 trials (16 hand images per condition × 12 combinations [one of the four conditions acted as the target and one of the remaining three conditions functioned as the distractor], e.g., *Self* vs. *Stranger*).

In Experiment 2a, there were two conditions: *Self* and *Other*. One of the 32 hand images (16 hand images from the *Self* condition and 16 hand images from the *Other* condition [including two strangers, eight images from each stranger]) was presented and remained on the screen until the participants responded (shown in Figure 1C). Participants were required to explicitly judge whether the displayed hand corresponded to their own or not using two arrow keys. Experiment 2a consisted of 96 trials with 32 hand images repeated thrice.

Notably, the different richness of stimuli (e.g., numbers of identities in the *Other* condition) were utilized in the classic and newly designed explicit tasks. To assess the potential effects of the richness of stimuli in the *Other* condition on the findings, we conducted Experiments 2b and 2c using the same paradigm as Experiment 2a but with a different richness of stimuli (e.g., numbers of identities) in the *Other* condition. Experiment 2b contained 128 trials with 16 hand images from the *Self* condition repeated four times and 32 hand images from the *Other* condition (including two strangers, 16 images from each stranger) repeated twice. Experiment 2c involved 192 trials with 16 hand images from the *Self* condition repeated six times and 48 hand images from the *Other* condition (i.e., friend, visually familiar individual, and stranger, with 16 hand images from one identity) repeated twice. Note that in all Experiments 2 series, the trials of *Self* were the same as those of *Other* (each 50%).

In Experiment 3, there were four conditions as in Experiment 1: *Self*, *Friend*, *Visually Familiar Individual*, and *Stranger*. One of 64 hand images (16 hand images from each of the four conditions) was presented and remained on the screen until participants responded (Figure 1D). Different from Experiment 2a, participants were required to explicitly judge whether the displayed hand corresponded to their own, their friend, visually familiar individual, or stranger with four arrow keys. Experiment 3 consisted of 192 trials with 64 hand images repeated thrice.

**Figure 2:**
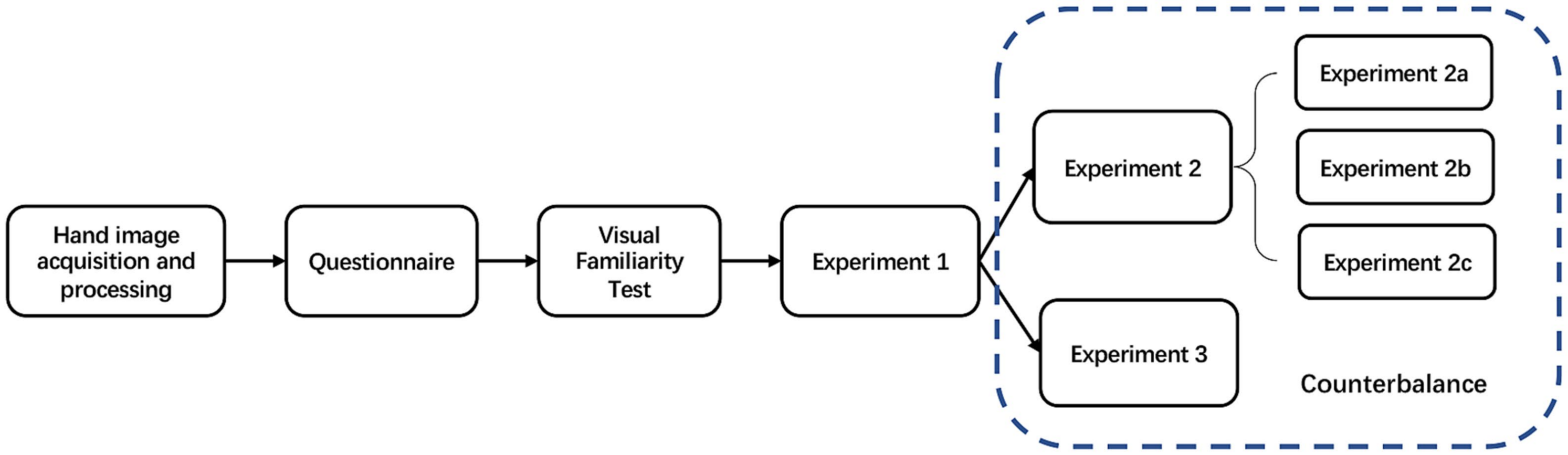
Experimental procedure. Three days before the experiments, hand images were photographed for each participant and their same-sex friends. On the day of the experiments, participants first completed a questionnaire. The experiments started with a practice phase to ensure that participants could recognize hand images from visually familiar individuals. To avoid participants assuming the purpose of the implicit task, they performed Experiment 1 before Experiments 2 (2a, 2b, and 2c) and 3. The order of experiments 2 and 3 was counterbalanced across participants.

To ensure participants did not assume the purpose of the implicit task, Experiment 1 was always conducted before Experiments 2 and 3 (shown in Figure 2). The order of Experiments 2 and 3 was counterbalanced across participants. Moreover, the response keys in Experiments 2 and 3 were also counterbalanced across participants.

Self-consciousness and explicit self-esteem were measured for each participant using the Self-Consciousness Scale (Fenigstein et al., 1975) and Rosenberg Self-Esteem Scale (Greenwald et al., 1998), respectively.

Participants were required to respond with their right index finger as accurately as possible. Experimental design details are given in Table 2.

**Table 2:** Details of the experimental design for each of the five experiments.

Exp	Task	Conditions (*N* of stimuli per individual/*N* of individuals)	Total *N* of trials (*N* of Trials per Condition)
1	Visual matching-to-sample	4 Self (16/1), Friend (16/1), Visually Familiar Individual (16/1), Stranger (16/1)	384 (96, 96, 96,96)
2a	Self-other discrimination	2 Self (16/1), Other [stranger (8/2)]	96 (48, 48)
2b	Self-other discrimination	2 Self (16/1), Other [stranger (16/2)]	128 (64, 64)
2c	Self-other discrimination	2 Self (16/1), Other [friend (16/1), visually familiar individual (16/1), stranger(16/1)]	192 (96, 96)
3	Identity discrimination	4 Self (16/1), Friend (16/1), Visually Familiar Individual (16/1), Stranger (16/1)	192 (48, 48, 48, 48)

Exp, experiment; *N*, number.

### 2.4. Data analyzes

Reaction times (RTs) of correct responses as well as accuracy (ACC) of responses were measured in all five experiments. For each participant, RT outliers, which differed from the mean of all trials by more than three SDs, were removed. To account for differences in the speed-accuracy trade-off across subjects, Efficiency Scores (ES) were computed by dividing ACC by RT for each condition, with higher ES indicating better performance. One-way repeated analysis of variance (ANOVA) was conducted with Identity (four levels in Experiments 1 and 3: *Self*, *Friend*, *Visually Familiar Individual*, and *Stranger*; two levels in Experiments 2 series: *Self* and *Other*) as the within-subject factor to compare participant performance under different conditions. We then applied *post hoc* tests, with adjustment for multiple testing using the *Bonferroni* method. Unless otherwise noted, all *p*-values were corrected.

Following established methodologies, we calculated the self-bias index (Amodeo et al., 2021) - the factor of interest - and examined the potential relationships of self-bias in implicit and explicit tasks. The self-bias index (Eq. 1) was calculated for each experiment, calculated for ACC, RT, and ES, respectively:


(1) $$Self–bias=\frac{self-stranger}{self+stranger}$$

## 3. Results

### 3.1. Experiment 3 (identity discrimination task, the newly designed explicit task)

**Figure 3:**
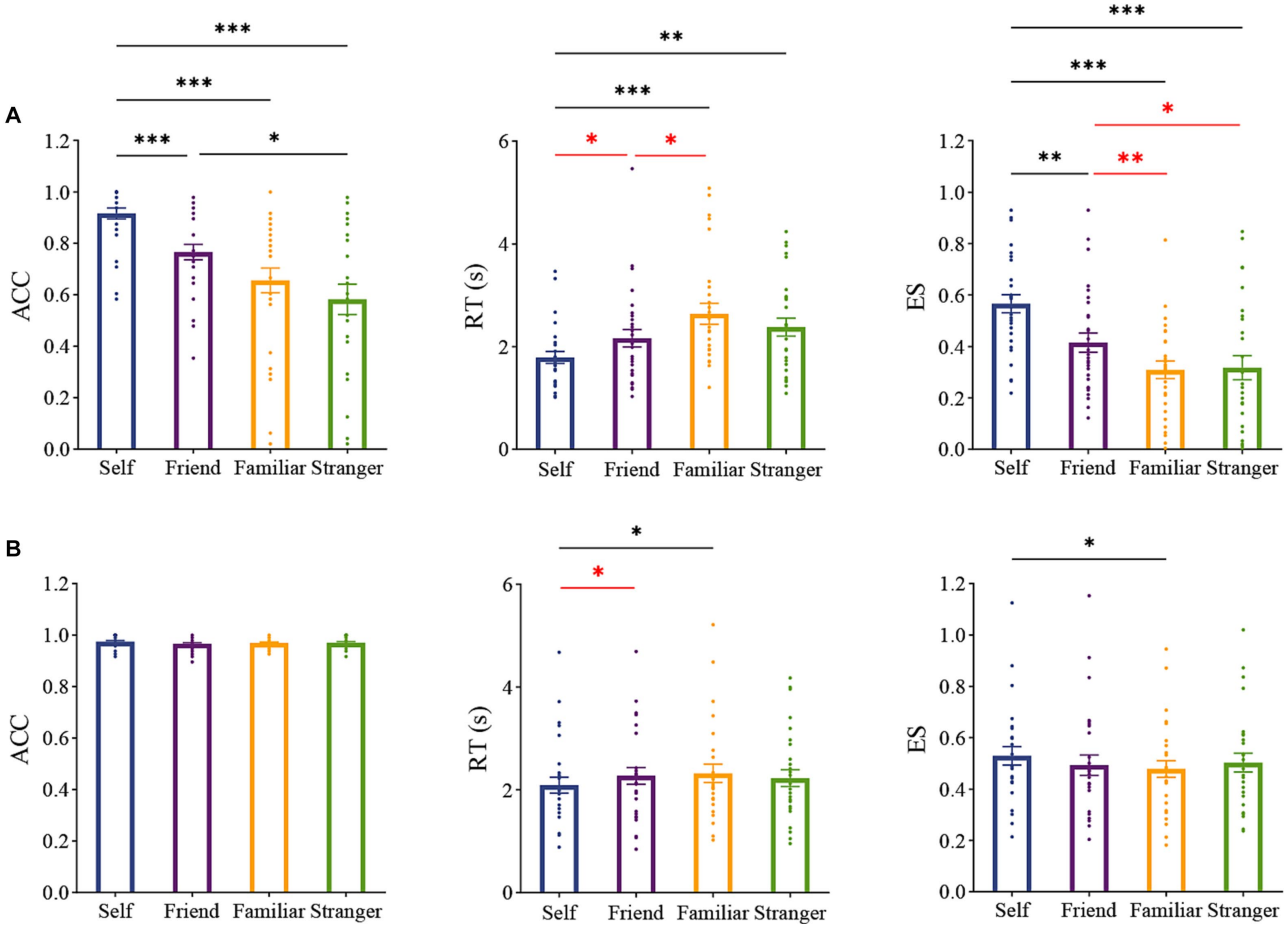
Results of Experiments 3 **(A)** and 1 **(B)**. Panels from left to right represent ACC, RT, and ES. Black * *p* < 0.05, ** *p* < 0.01, *** *p* < 0.001 corrected. Red * *p* < 0.05, ** *p* < 0.01 uncorrected. Error bars indicate standard error.

To test our hypothesis, we designed a novel explicit task, in which participants were required to judge the identity of a hand image rather than whether it was their own or not. The ANOVA results showed that Identity had significant effects on ACC [*F* (3,84) = 15.319, *p* < 0.001, partial η2 = 0.354], RT [*F* (3,84) = 7.483, *p* < 0.001, partial η2 = 0.211], and ES [*F* (3,84) = 17.218, *p* < 0.001, partial *η*^2^ = 0.381] (Figure 3A). Post-hoc analyzes revealed that ACC was significantly (*p* < 0.001) higher in the *Self* condition (0.917) than in the *Friend* (0.766), *Visually Familiar Individual* (0.657), and *Stranger* conditions (0.583). Participants also responded significantly faster in the *Self* condition than in the other three conditions (vs. *Friend*: *p* = 0.046, uncorrected; vs. *Visually Familiar Individual*: *p* < 0.001; vs. *Stranger*: *p* = 0.005). Consequently, ES was significantly higher in the *Self* condition than in the other three conditions (vs. *Friend*: *p* = 0.006; vs. *Visually Familiar Individual*: *p* < 0.001; vs. *Stranger*: *p* < 0.001). Thus, these findings suggested the existence of self-advantage in the newly designed explicit task. Participants also performed better in the *Friend* condition than in the *Visually Familiar Individual* (*p* = 0.009) and *Stranger* conditions (*p* = 0.035).

### 3.2. Experiment 1 (visual matching-to-sample task, the classic implicit task)

Next, we looked into the results from Experiment 1 (Figure 3B), in which the same implicit task as those in the previous studies (Frassinetti et al., 2010; Richetin et al., 2012) was conducted.

We conducted one-way repeated ANOVAs with Identity as the within-subject factor. No significant main effects of Identity on ACC [*F* (3,84) = 0.630, *p* = 0.597, partial η^2^ = 0.022] or ES (*F* (3,84) = 2.063, *p* = 0.111, partial η2 = 0.069) were found. However, there was a significant main effect of Identity on RTs [*F* (3,84) = 3.302, *p* = 0.024, partial η2 = 0.105]. *Post hoc* tests further revealed that participants responded faster in the *Self* condition than in the *Friend* (*p* = 0.028, uncorrected) and *Visually Familiar Individual* (*p* = 0.020) conditions (Figure 3B). Participants also tended to respond faster in the *Self* condition than in the *Stranger* condition (*p* = 0.172, uncorrected). These findings indicated self-advantage, consistent with previous findings (Ferri et al., 2011; Conson et al., 2015; Candini et al., 2016; Fukui et al., 2020).

To facilitate the comparison with Experiment 3 and 2 series (explicit tasks), in which there were two conditions: *Self* and *Other*, we aggregated participants’ performance in all non-self conditions (i.e., *Friend*, *Visually Familiar Individual,* and *Stranger*) and then compared this to their performance in the *Self* condition. We found that participants responded faster in the *Self* condition than in the aggregated *Other* condition (RTs: *p* = 0.017; ES: *p* = 0.048). Again, these results suggest the existence of self-advantage in the implicit task.

### 3.3. Experiment 2 series (self-other discrimination task, the classic explicit task)

In the Experiment 2 series, we conducted the same explicit tasks as previous studies (Ferri et al., 2011; Richetin et al., 2012) with different sets of *Other* stimuli.

**Figure 4:**
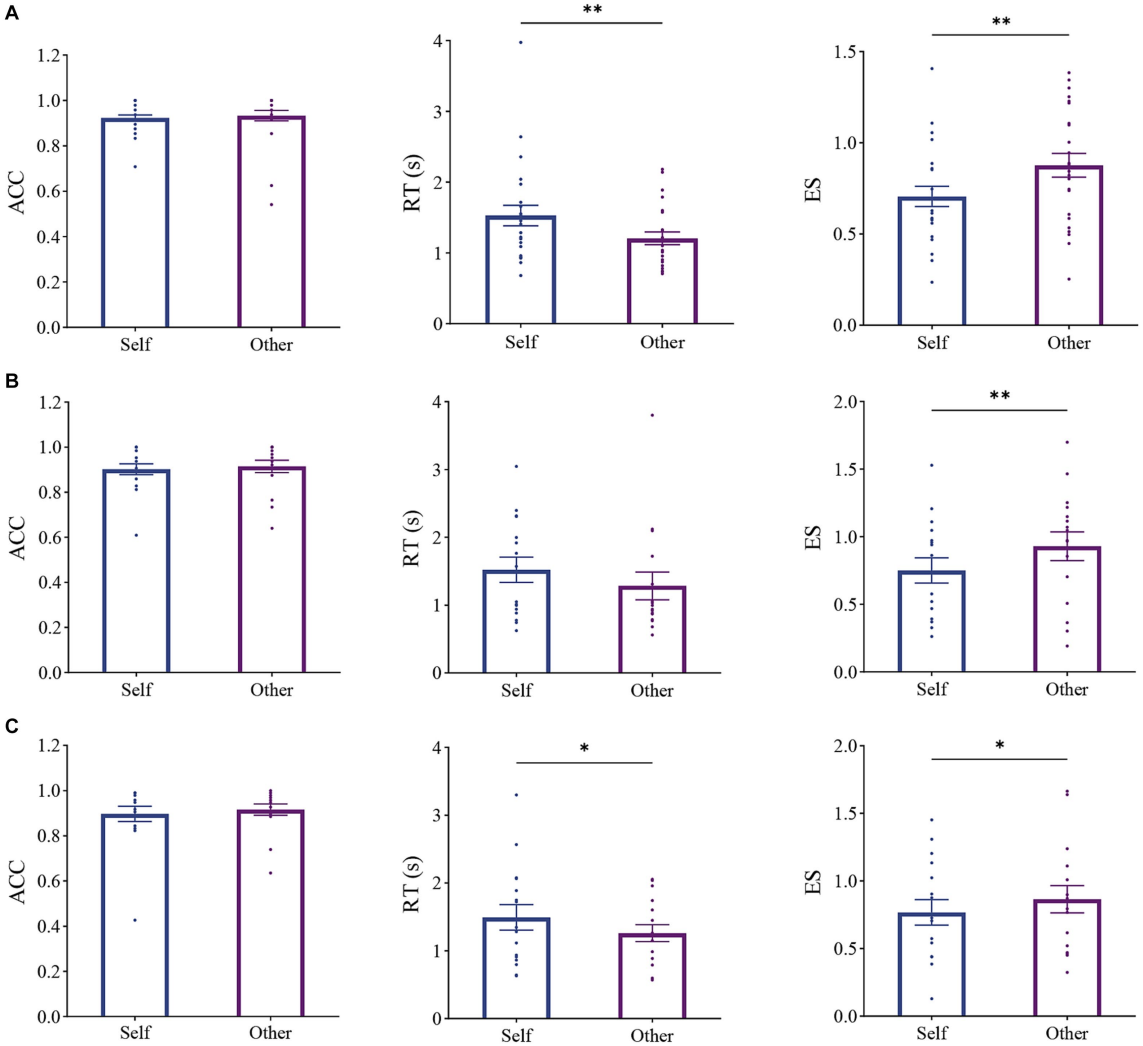
Results of Experiments 2a **(A)**, 2b **(B)**, and 2c **(C)**. Panels from left to right represent ACC, RT, and ES; * *p* < 0.05, ** *p* < 0.01, corrected. Error bars indicate standard error.

First, we looked into the results in Experiment 2a, which had the same *Other* stimulus settings as the prior studies. ANOVAs were also conducted on participants’ ACC, RTs, and ES (Table 3). We found that ACCs in the *Self* and *Other* conditions were similar. The main effects of Identity on RTs and ES were significant or approximately significant: participants performed worse in the *Self* condition than in the *Other* condition, indicating self-disadvantage in the explicit task (Figure 4A), in line with previous findings (Ferri et al., 2011; Conson et al., 2015; Candini et al., 2016; Fukui et al., 2020).

**Table 3:** ANOVA results of Experiment 2 series, with identity (self and other) as a within-subject factor.

	df (df1, df2)	ACC			RTs			ES		
		F	*p*	partial *η*^2^	F	*p*	partial η2	F	*p*	partial *η*^2^
Exp 2a	(1, 23)	0.108	0.746	0.005	9.099	0.006**	0.283	9.401	0.005**	0.290
Exp 2b	(1, 15)	0.103	0.753	0.007	4.057	0.062	0.213	9.455	0.008**	0.387
Exp 2c	(1, 15)	0.489	0.495	0.032	6.233	0.025*	0.294	5.832	0.029*	0.280

*p < 0.05, **p < 0.01.

Notably, different numbers of identities in the *Other* condition were utilized in the newly designed (i.e., one friend, one visually familiar individual, and one stranger) and classic (i.e., two strangers) explicit tasks. To investigate whether stimulus richness induced self-disadvantage but not self-advantage in the classic explicit task, we conducted Experiments 2b and 2c. Especially, Experiment 2c consisted of the same stimulus set as Experiment 1. Similar results to those in Experiment 2a were found (Table 3; Figures 4B,C). That is, even though more non-self stimuli were used, participants still showed poorer performance in the *Self* condition than in the *Other* condition, indicating that the self-disadvantage found in the classic explicit task could not be explained by the stimulus set.

### 3.4. Correlations between performance across behavioral tasks

**Figure 5:**
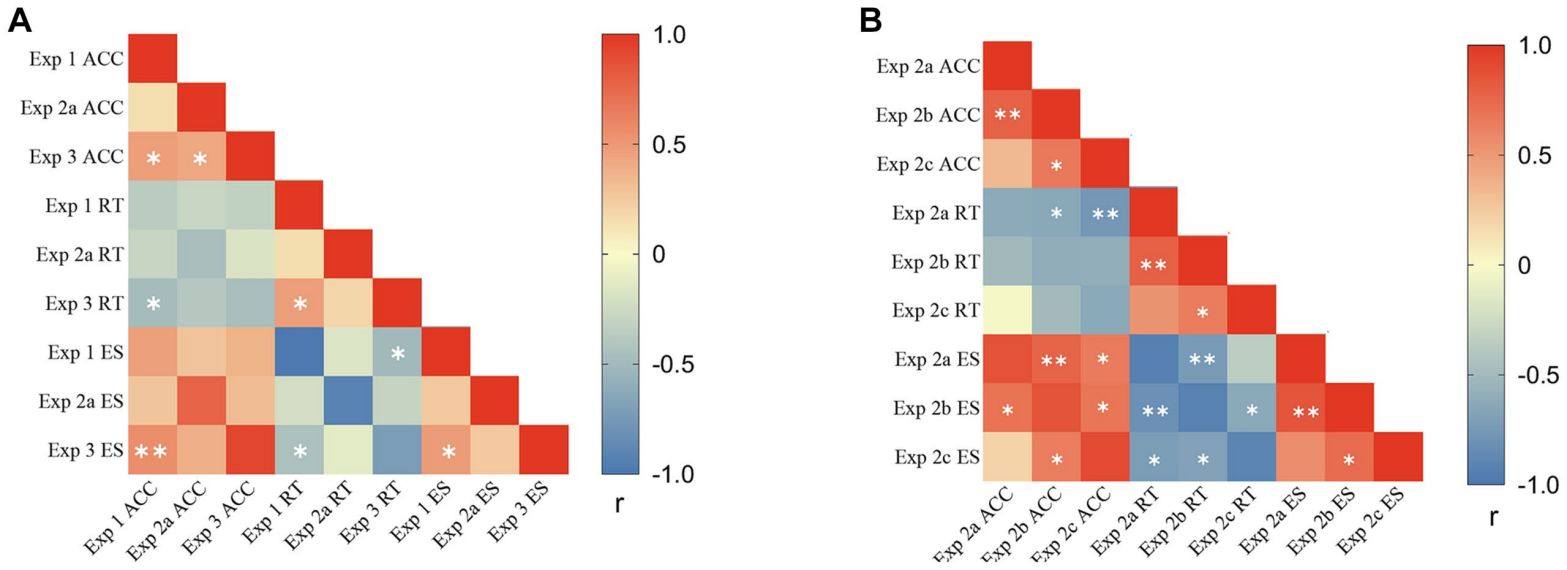
Correlations of self-bias indices among Experiments 1, 2a, and 3 **(A)** and among Experiment 2 series **(B)**; * *p* < 0.05, ** *p* < 0.01, and *** *p* < 0.001; two-tailed Pearson correlation.

To further evaluate the relationship between the newly designed explicit task (Experiment 3) and the classic tasks (explicit: Experiment 2a; implicit: Experiment 1), we conducted Pearson correlations (two-tailed) on the self-bias indices based on ACC, RT, and ES, respectively. Notably, we found significant correlations between Experiment 3 and 1&2a, with no correlations between Experiments 1 and 2a (Figure 5A). These findings suggest a strong link between self-advantage found in the classic implicit task and the newly designed explicit task, but no link between self-disadvantage found in the classic explicit task and self-advantage found in the classic implicit task.

We further explored the relationship among the three self-other discrimination experiments (Experiments 2a, 2b, and 2c). Pearson correlations (two-tailed) were conducted on the self-bias indices based on ACC, RT, and ES, respectively. Results showed significant correlations among the three experiments (Figure 5B). Again, these results indicated that the richness of non-self stimuli did not affect the self-disadvantage found in the classic explicit experiments.

### 3.5. Correlations between performance in behavioral tasks and reported self-consciousness and explicit self-esteem

**Figure 6:**
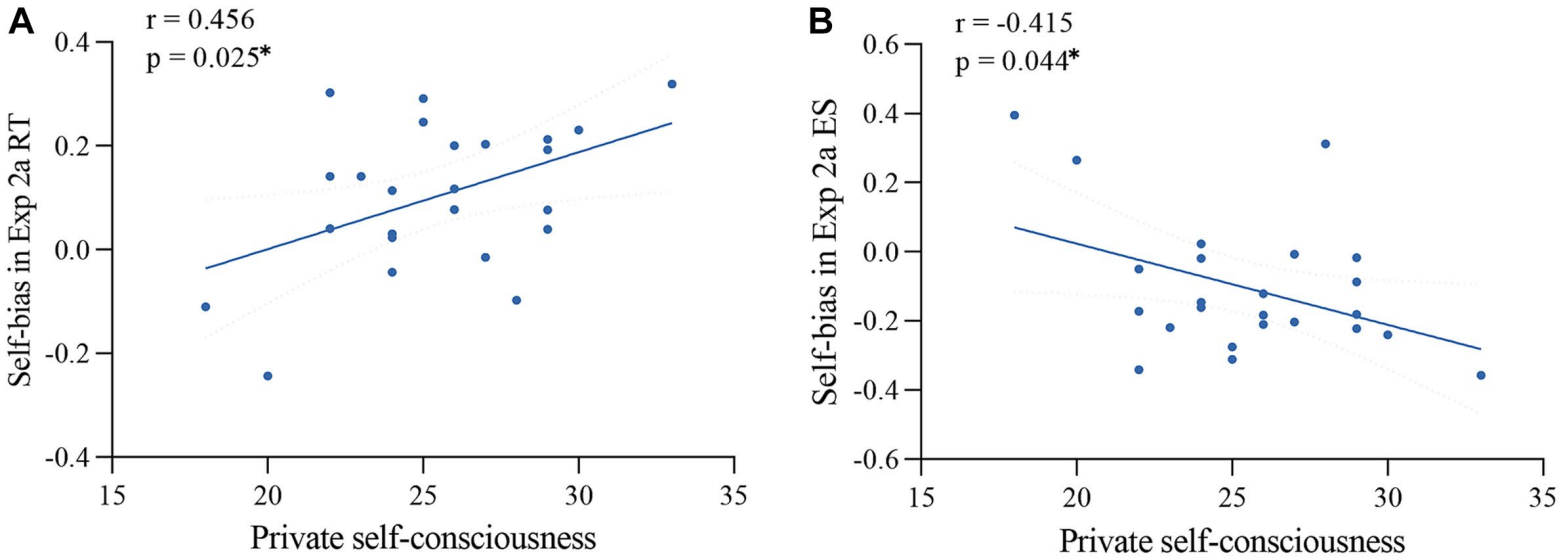
Correlations between self-bias in Experiment 2a [**(A)**: RT, **(B)**: ES] and private self-consciousness scores. Dashed lines indicate 95% confidence intervals. * *p* < 0.05; two-tailed Pearson correlation.

Using two-tailed Pearson correlations (see Table 4), we found that private self-consciousness was significantly correlated with self-bias measured in Experiment 2a (Figure 6), with higher private self-consciousness scores indicating greater self-disadvantage. No significant correlations were found between the questionnaire measures and self-advantage in the implicit and newly designed explicit tasks.

**Table 4:** Correlation coefficients among self-bias indices of Experiments 1, 2a, and 3, and questionnaire scores.

	ACC			RT			ES		
	Exp 1	Exp 2a	Exp 3	Exp 1	Exp 2a	Exp 3	Exp 1	Exp 2a	Exp 3
Private self-consciousness	−0.10	−0.20	−0.31	0.18	0.46*	0.33	−0.18	−0.41*	−0.26
Public self-consciousness	−0.24	0.21	−0.16	−0.06	0.26	−0.03	0.03	0.09	−0.12
Social anxiety	−0.21	0.03	−0.26	0.05	0.40	0.14	−0.07	−0.27	−0.21
Self-esteem	−0.03	0.30	0.12	−0.14	−0.21	−0.20	0.13	0.29	0.10

*p < 0.05, two-tailed Pearson correlation.

## 4. Discussion

We designed a novel explicit task (Experiment 3) and performed the same explicit (Experiment 2 series) and implicit (Experiment 1) tasks as in previous studies to investigate whether the self-hand can induce recognition advantage and, if so, whether such advantage could be found in both implicit and explicit self-hand recognition. Results showed self-advantage in the implicit task and self-disadvantage in the explicit task in self-hand recognition, consistent with previous findings (Ferri et al., 2011; Conson et al., 2015; Candini et al., 2016; Fukui et al., 2020). Importantly, with the newly designed explicit task, we found clear and robust evidence of self-hand recognition advantage, thus providing new insights into the long-standing inconsistencies in previous studies. Below, we discuss the importance of these findings for understanding self-advantage.

For self-related information (e.g., faces), recognition advantage exists at both the explicit and implicit levels (Devue et al., 2007; Keyes and Brady, 2010; Malaspina et al., 2019). However, findings related to self-body recognition are inconsistent at these two levels. That is, self-advantage for self-face and self-body is strongly correlated in implicit tasks but not explicit tasks (Malaspina et al., 2019). How can this self-bias puzzle be explained?

In previous studies, participants were required to make “self” or “non-self” judgments in the explicit tasks but match “self” and “non-self” stimuli to sample stimuli in the implicit tasks. In this case, the method of exclusion (it is not mine) may be applied in the explicit tasks, whereas the method of direct selection (it is the sample) may be applied in the implicit tasks. Obviously, it is easier to judge that it is not A (e.g., self) than it is A (Mynatt et al., 1977). Thus, the self-disadvantage found in explicit experiments may arise from the advantage of judging “it is not mine” versus “it is mine.” To test this possibility, we asked participants to perform the same actions on both self- and non-self hands: i.e., to which individual does the stimulus belong — self, friend, visually familiar individual, or stranger? With this newly designed explicit task, we did find self-advantage same as that found in the implicit task, thereby confirming our hypothesis. Of note, self-advantage was consistently found across all three measures (i.e., ACC, RTs, and ES). These findings suggest the existence of self-advantage in explicit hand recognition.

In previous implicit and explicit studies (Aranda et al., 2010; Ferri et al., 2011; Frassinetti et al., 2011; Candini et al., 2016; Conson et al., 2017; Fukui et al., 2020; Kuroki and Fukui, 2020), to familiarize participants with the task, it has been a common procedure to include a practice phase, which utilized the same stimuli as the main experiments and exposed participants to both self-relevant and other-relevant images prior to the main experiments. As a result, compared with previous studies, even if the brief preview phase in the present study had any impact on subsequent experiments, it would likely be minimal and primarily limited to the implicit task. Note that our results showed self-advantage in the implicit task, suggesting that the brief preview phase in the present study did not cause significant differences between our findings and previous implicit studies (Frassinetti et al., 2011; Richetin et al., 2012; De Bellis et al., 2017; Malaspina et al., 2018). Furthermore, it is noteworthy that our main objective was not to re-examine the well-documented self-advantage observed in implicit tasks. Instead, the main purpose of the present study was to develop a novel explicit task and demonstrate whether the inconsistency between self-body bias in explicit and implicit tasks is related to behavioral strategies in explicit tasks. The brief preview phase in the present study was similar to the practice phase in previous studies (Ferri et al., 2011; Frassinetti et al., 2011; Candini et al., 2016; Conson et al., 2017; Fukui et al., 2020; Kuroki and Fukui, 2020). Indeed, our results in the classic explicit task showed self-disadvantage, aligning with previous findings, further confirming that any potential impact of the brief preview phase in our study on the explicit task, if present, would be comparable to previous studies. More importantly, the impact of stimulus exposure was comparable between our newly designed explicit task and the classic explicit tasks. Therefore, it is unlikely that the observed dissociation in self-bias during the newly designed and classic explicit tasks could be solely attributed to the mere presentation of self-hands before the experimental tasks.

Furthermore, our results showed significant correlations between the self-bias indices in the classic implicit task and newly designed explicit task, indicating potentially shared strategies or even neural pathways involved in implicit and explicit processing. Note that our findings could not preclude the possibility that implicit and explicit self-body recognition pathways may be distinct, despite the presence of self-advantage at both implicit and explicit levels. Further studies employing neuroimaging approaches may elucidate the underlying neural mechanisms.

The same stimulus set used in the implicit task was presented in the newly designed explicit task. To exclude the possibility that the inconsistency between the classic explicit and implicit tasks may be caused by differences in the stimulus set (e.g., the number of identities enrolled in tasks), we modified the classic explicit task and conducted two experiments (Experiments 2b and 2c) using the same paradigm as Experiment 2a, except for the stimulus set. In addition to self-hand images, there were eight images from each of the two strangers in Experiment 2a (equal number of stranger and self images), 16 images from each of the two strangers in Experiment 2b (equal number of images from each individual), and 64 images from friend, stranger, and visually familiar stranger (i.e., same stimulus set as Experiments 1 and 3) in Experiment 2c. We found similar results in all three self-other discrimination experiments as well as significant correlations among them, suggesting that stimulus richness was not the cause for self-disadvantage in self-hand recognition. These results are consistent with previous findings showing that if self-other discrimination tasks are conducted to assess self-body recognition, no self-advantage is found (Frassinetti et al., 2011; Richetin et al., 2012), or even self-disadvantage can be detected (Ferri et al., 2011; Conson et al., 2015; Candini et al., 2016; Kuroki and Fukui, 2020), regardless of the type of stimuli used in the explicit task.

We did not find significant correlations between scores in self-related questionnaires (i.e., private self-consciousness and self-esteem) and self-advantage found in the implicit and newly designed explicit tasks. While several studies have found relationships between scores acquired from self-related questionnaires and self-bias measured by the implicit and explicit tasks (Richetin et al., 2012), not all studies have identified such relationships (for exceptions, see Williams et al., 2018; Jiang et al., 2019; Nijhof et al., 2020; Amodeo et al., 2021). The self-concept has been considered ambiguous and abstract, posing challenges for accurate measurement through questionnaires (Grace and Cramer, 2003). The reliability of questionnaire measures has been questioned due to inconsistent results (Levine et al., 2006). As a result, it may not be surprising that no significant correlations were found between the questionnaire measures and self-advantage in the implicit and newly designed explicit tasks. Note that private self-consciousness scores were correlated with the self-bias indices measured in the classic explicit task (Experiment 2a). To the best of our knowledge, previous studies have mainly found relationships between self-esteem but not self-consciousness measured with questionnaires and self-bias in behavioral tasks (Richetin et al., 2012). Therefore, the reliability of this correlation may need to be re-tested in future studies, which may also be designed to capture the underlying basis of this relationship.

## 5. Conclusion

Taken together, we designed a novel explicit paradigm to detect self-advantage in hand recognition. By using this task and a series of classic explicit and implicit tasks, we revealed the reason for inconsistencies between implicit and explicit results in previous studies: participants may utilize different strategies in classic explicit and implicit tasks. Importantly, we obtained compelling evidence that the self-hand recognition advantage exists at both the explicit and implicit levels, and that the self-advantage processing by these two pathways may be linked. Our findings reveal the extent to which self-bias is conserved across self-related information. We anticipate that these results will deepen our understanding of self-bias and open a new avenue for studying self-bias using self-body stimuli, which are less affected by social information carried by faces (e.g., emotion and personality) (Lee et al., 2008; Quevedo et al., 2018).

## Data availability statement

The raw data supporting the conclusions of this article will be made available by the authors, without undue reservation.

## Ethics statement

The studies involving human participants were reviewed and approved by Institutional Review Board of Institute of Biophysics. The patients/participants provided their written informed consent to participate in this study.

## Author contributions

SX and MZ: conceptualization, methodology, validation, data collection, investigation, formal analysis, writing – original draft, writing – review, and editing. LY: data collection. NL: conceptualization, methodology, writing – original draft, writing – review and editing, supervision, and project administration. All authors contributed to the article and approved the submitted version.

## Funding

This work was supported by STI2030-Major Projects (Grant nos. 2021ZD0200200 and 2021ZD0204200), the National Natural Science Foundation of China (Grant no. 32071094), Strategic Priority Research Program of Chinese Academy of Science (XDB32020207), Chinese Academy of Sciences (QYZDB-SSW-SMC033).

## Conflict of interest

The authors declare that the research was conducted in the absence of any commercial or financial relationships that could be construed as a potential conflict of interest.

## Publisher’s note

All claims expressed in this article are solely those of the authors and do not necessarily represent those of their affiliated organizations, or those of the publisher, the editors and the reviewers. Any product that may be evaluated in this article, or claim that may be made by its manufacturer, is not guaranteed or endorsed by the publisher.

## Footnotes

^1^http://psychtoolbox.org/
